# Lack of impact of iodinated contrast media on kidney cell-cycle arrest biomarkers in critically ill patients

**DOI:** 10.1186/s12882-018-1091-2

**Published:** 2018-11-06

**Authors:** Emmanuelle Rouve, Karim Lakhal, Charlotte Salmon Gandonnière, Youenn Jouan, Laetitia Bodet-Contentin, Stephan Ehrmann

**Affiliations:** 1 Medecine Intensive Reanimation, CIC 1415, CRICS-Triggersep network, CHRU de Tours, Tours, France; 2 Reanimation chirurgicale polyvalente, service d’anesthesie-reanimation, Hôpital Laënnec, centre hospitalier universitaire, Nantes, France; 3 Faculté de médecine, Université de Tours, Tours, France

**Keywords:** Acute kidney injury, Iodinated contrast media, Biomarkers, TIMP-2, IGFBP-7, Iohexol, Iobitridol, Iopromide

## Abstract

**Background:**

Iodinated contrast media may contribute to acute kidney injury. However, several recent works suggest that this toxicity is minimal in the clinical setting. Recently, urinary G1 cell-cycle arrest proteins *tissue inhibitor of metalloproteinase 2* (TIMP-2) and *insulin like growth factor binding protein 7* (IGFBP-7) were identified as highly sensitive and specific biomarkers for early detection of kidney aggression. The impact of contrast administration on those biomarkers has not been specifically evaluated but could provide clues about the toxicity of contrast media. This study aimed at measuring changes in TIMP-2 and IGFBP-7 urinary concentrations before and after a contrast-enhanced computed tomography in critically ill patients.

**Methods:**

77 patients were included in a prospective observational cohort study. Urinary [TIMP -2]·[IGFBP-7] was measured before, 6 and 24 h after contrast infusion. Urine output and serum creatinine were followed 3 days.

**Results:**

Median [TIMP-2]·[IGFBP-7] was 0.06 [interquartile range 0.04;0.26], 0.07 [0.03;0.34] and 0.10 [0.04;0.37] (ng/mL)^2^/1000 respectively before, 6 and 24 h after contrast infusion. Individual changes from baseline were − 0.01 [− 0.11;0.11] and 0.00 [− 0.10;0.09] (ng/ml)^2^/1000 at 6 and 24 h. These changes were not higher among the patients increasing their Kidney Disease Improving Global Outcome (KDIGO) classification within 3 days after contrast infusion (*n* = 14 [18%] based on creatinine criterion only, *n* = 42 [55%] based on creatinine and urine output).

**Conclusions:**

Changes in [TIMP-2]·[IGFBP-7] urinary concentration after contrast-enhanced computed tomography were insignificant, suggesting minimal kidney aggression by modern iodinated contrast media.

## Background

Acute kidney injury (AKI) is common among critically ill patients [1]. Even mild forms are associated with significant mortality and morbidity [2, 3]. Among the numerous causes of AKI in the intensive care unit (ICU), the toxicity of iodinated contrast media (CM) has been considered, for long, to be an important contributor, so called contrast-associated AKI (CA-AKI) [2]. Indeed, the nephrotoxicity of iodinated CM is undisputed in experimental studies [4]. However, in the clinical setting, several recent works suggest that, using modern low osmolarity iodinated CM, the incidence of AKI following exposition to CM is not higher than in matched non-exposed patients [5–9]. In other words, there is increasing evidence of a minimal nephrotoxicity directly attributable to iodinated CM in the clinical setting of multifactorial renal aggression in the ICU, but also among general ward patients and in the emergency department [5–12]. Early AKI detection is challenging as diagnosis is delayed until serum creatinine concentration rises and/or urine output decreases. Such delayed AKI diagnosis makes it difficult to assess the causal implication of CM among other renal aggressions. Performing a randomized trial testing CM versus placebo, albeit desirable to evaluate CM implication without any bias, would be ethically, at best, very difficult to perform. Thus, other methodologies, such as kidney aggression biomarkers analysis may be of value to complete the evidence provided by epidemiologic studies. Recently, in ICU patients, cell-cycle arrest biomarkers were identified as very promising for early detection of kidney aggression: the combination of tissue inhibitor of metalloproteinases-2 (TIMP-2) and insulin-like growth factor binding protein-7 (IGFBP-7) showed high sensitivity and specificity, outperforming other biomarkers for early detection of AKI [13, 14]. To the best of our knowledge, the performance of [TIMP-2]·[IGFBP-7] has not been specifically evaluated in toxic AKI, despite some common pathophysiologic pathways shared among all AKI etiologies. Thus, quantifying cell-cycle arrest biomarkers before and after iodinated CM infusion as a direct evaluation of kidney aggression could be a means to confirm the recent, creatinine-based, epidemiologic evidence questioning the clinical relevance of CA-AKI. Furthermore, in the clinical setting of multifactorial kidney aggression, evaluating the effect of iodinated CM on those biomarkers is also important.

The aim of this preliminary study was to measure changes in TIMP-2 and IGFBP-7 urinary concentrations after iodinated CM-enhanced computed tomography in ICU patients.

## Methods

We prospectively included adult critically ill patients with a urinary catheter undergoing a first intravenous iodinated CM-enhanced computed tomography. Patients were not included in case of anuria or other reasons preventing urine analysis (e.g. urine mixed with drainage fluid). The study protocol was approved by the ethics board of the French intensive care society (CE SRLF 14–16). In accordance with national regulations, patients and/or relatives were informed about the study and could decline participation, written consent was waived. Presentation follows the STROBE statement on reporting observational studies according to the EQUATOR guidelines [15].

Three urine samples were collected from the external collection bag attached to the urinary catheter. The first sample was drawn few minutes before the transfer to the imaging facility (baseline), the second 6 h after and the third 24 h after the infusion of iodinated CM. Within 1 h after collection, urine samples were centrifuged 10 min at 2600 g then supernatants were immediately stored at − 20 °C. At study completion, samples were thawed immediately prior to analysis. IGFBP-7 and TIMP-2 were simultaneously measured using the NephroCheck® immunoassay bench-top device (Astute Medical, San Diego, USA), blind of clinical data. The device displays the immunoassay results as a single dimensionless numerical value ([ng/mL]^2^/1000, units not further reported in the manuscript) representing TIMP-2 urinary concentration multiplied by IGFBP-7 urinary concentration ([TIMP-2]·[IGFBP-7]).

Clinical data were prospectively recorded in patients’ charts: patient demographics, prior health history, serum creatinine concentration before the current admission (during the preceding year, outside of any AKI) and last serum creatinine concentration before CM infusion (baseline serum creatinine concentration), main ICU admission diagnosis, reason for computed tomography, type and volume of CM infused.

Daily data from inclusion to the third day after CM infusion were also collected: serum creatinine, urine output and nephrotoxic drug administrations. The occurrence of AKI was evaluated according to the Kidney Disease Improving Global Outcome (KDIGO) guidelines [2]: for instance, stage 1 was defined as a 1.5–1.9 times increase or ≥ 26.5 μmol/l increase in serum creatinine as compared with baseline serum creatinine concentration and/or a urine output of < 0.5 ml/kg/h for 6–12 h.

### Outcomes

The primary objective of the study was to calculate individual changes in [TIMP-2]·[IGFBP-7] from baseline to 6 and 24 h after iodinated CM infusion. To the best of our knowledge, no cutoff has been proposed for clinically significant changes in [TIMP-2]·[IGFBP-7] over time. We chose the 0.3 cutoff as representing a significant increase in [TIMP-2]·[IGFBP-7] after CM infusion compared to baseline and calculated the proportion of patients showing such an increase. We also calculated the proportion of patients with [TIMP-2]·[IGFBP-7] values above validated cut-offs (0.3, 1.0 and 2.0) at baseline, 6 h and 24 h after CM infusion [14, 16, 17].

As a secondary objective, changes in [TIMP-2]·[IGFBP-7] were compared between patients developing CA-AKI, defined as a KDIGO score increase within 3 days of iodinated CM infusion. As ongoing AKI at the time of CM infusion may represent a confounding factor, analysis was also carried out among the subgroup of patients without AKI at the time of CM infusion.

### Statistical analysis

Qualitative variables were reported as absolute count (percentage) and quantitative variables as median [first; third quartiles]. This descriptive study did not require a formal sample size calculation. However, to detect a 0.3 increase in [TIMP-2]·[IGFBP-7] (value considered as clinically significant), with a power of 80%, an alpha risk of 5%, as compared to no change in [TIMP-2]·[IGFBP-7] after iodinated CM infusion (zero increase), considering a standard deviation of 0.9 for [TIMP-2]·[IGFBP-7], 71 patients needed to be included. Importantly, this preliminary study was not powered to evaluate [TIMP-2]·[IGFBP-7] as a predictive marker of CA-AKI but may give information for future sample size calculations in this regard. Statistical comparisons used non-parametric tests (paired Wilcoxon signed rank test to analyze individual changes in [TIMP-2]·[IGFBP-7] within patients, Mann Whitney test to compare patients developing or not CA-AKI, Chi^2^ or Fisher exact test to compare categorical variables) with Medcalc® software (Ostende, Belgium). Analysis was performed in the whole study population and among the subgroup of patients without ongoing AKI at inclusion. [TIMP-2]·[IGFBP-7] was not available at 24 h after inclusion and thus not analyzed for 5 patients due to early discharges or death and 2 missing samples. No other data were missing. A *p* value lower than 0.05 was considered significant.

## Results

From April 2014 to March 2016, 77 patients were included (Table 1). Most patients presented with acute respiratory failure, coma and sepsis at ICU admission. A history of chronic kidney disease was present for 5 patients (7%). CM infusion was performed 48 h [18; 183] after ICU admission. Seventeen patients (27%) were exposed to additional nephrotoxic medication the day before CM infusion. At baseline, 42 patients (55%) had no AKI, 20 (26%) had KDIGO stage 1, 13 (17%) stage 2 and 2 (3%) stage 3 AKI. Thirty-eight patients (49%) received fluid loading immediately before CM administration. No other specific CA-AKI prevention measure was implemented. Twenty patients (26%) were exposed to additional nephrotoxic drugs the day of CM infusion.

**Table 1:** Patients’ characteristics

	*n* = 77
Age (years)	64 [53; 70]
Female gender	19 (25%)
Comorbidities	
Chronic kidney disease	5 (7%)
Hypertension	26 (34%)
Diabetes	14 (18%)
Cancer	26 (34%)
Cirrhosis	3 (4%)
Congestive heart failure	4 (5%)
Chronic obstructive pulmonary disease	1 (1%)
Main ICU admission diagnosis	
Acute respiratory failure	26 (34%)
Coma	19 (25%)
Sepsis or septic shock	12 (16%)
Other shock	8 (10%)
Exacerbation of chronic respiratory failure	3 (4%)
Cardiac arrest	2 (3%)
Multiple organ failure	2 (3%)
Other	5 (6%)
SAPS^a^ II at admission	46 [35; 56]
Serum creatinine concentration at inclusion (μmol/L)	69 [48; 109]
Computed tomography explored area^b^	
Brain	29 (38%)
Thorax	40 (52%)
Abdomen	29 (38%)
Pelvis	26 (34%)
Soft tissue/limbs	1 (1%)
Spine	1 (1%)
Iodinated contrast medium infused	
Iohexol 300 mg/mL iodine	3 (4%)
Iohexol 350 mg/mL iodine	26 (34%)
Iobitridol 350 mg/mL iodine	26 (34%)
Iopromide 370 mg/mL iodine	17 (22%)
Iomeprol 350 mg/mL iodine	1 (1%)
Iomeprol 400 mg/mL iodine	4 (5%)
Volume of iodinated contrast medium infused (mL)	96 [60; 120]

Qualitative variables are reported as count (percentage) and quantitative variables as median [first; third quartiles]

^a^simplified acute physiology score

^b^percentages add up to more than 100% because a given patient may have had several anatomic regions explored within one imaging procedure

### Change in [TIMP-2]·[IGFBP-7] in the whole population

**Fig. 1:**
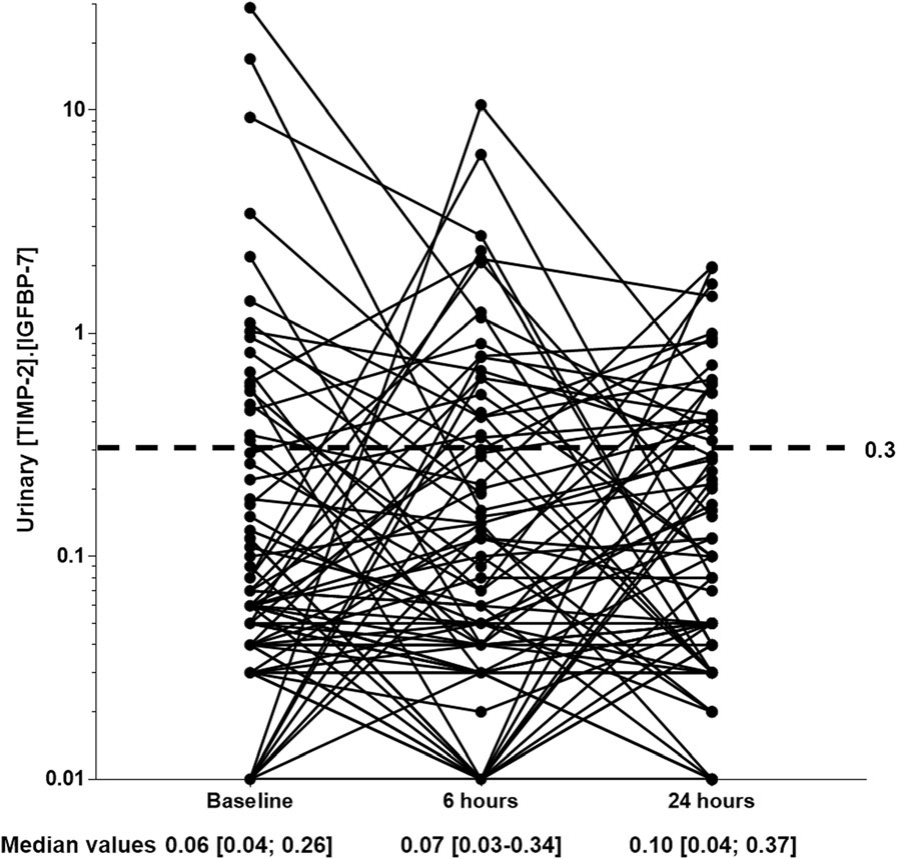
Urinary concentrations of [TIMP-2]**·**[IGFBP-7]. Logarithmic scale on the Y axis. A [TIMP-2]·[IGFBP-7] (NephroCheck®) value higher than 0.3 is considered significant. [TIMP-2]·[IGFBP-7] was not available at 24 h for 5 (6%) patients. No other data were missing. TIMP-2: tissue inhibitor of metalloproteinases-2; IGFBP-7: insulin-like growth factor binding protein-7

Baseline median [TIMP-2]·[IGFBP-7] value was 0.06 [0.04; 0.26]. Six hours after CM infusion, the median [TIMP-2]·[IGFBP-7] value was 0.07 [0.03–0.34]. The median individual change in [TIMP-2]·[IGFBP-7] value from baseline to 6 h later was − 0.01 [− 0.11; 0.11], not significantly different from 0 (*p* = 0.72). Individual changes in [TIMP-2]·[IGFBP-7] values are represented in Fig. 1.

**Fig. 2:**
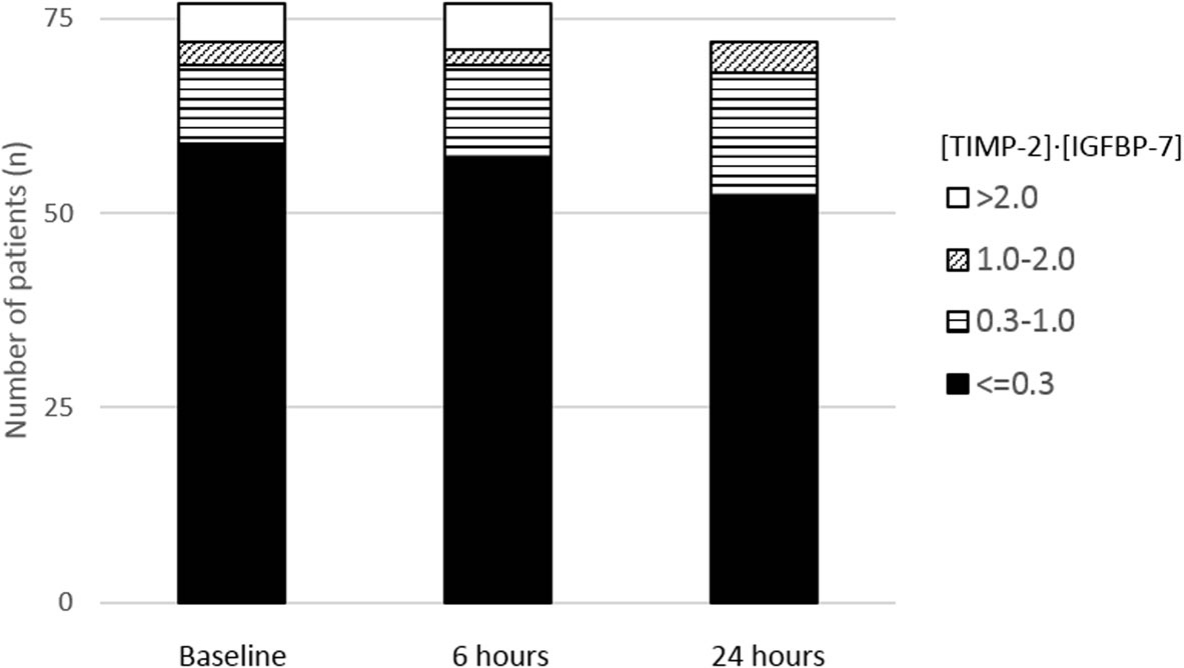
Distribution of urinary concentrations of [TIMP-2]·[IGFBP-7]. At baseline, i.e., before contrast media infusion, 59 (77%) patients had [TIMP-2]·[IGFBP-7] values ≤0.3; 10 (13%) had values]0.30–1.0]; 3 (4%) had values]1.0–2.0]; 5 (6%) had values > 2.0. Six hours and 24 h after contrast media infusion, distributions were 57 (74%), 12 (15%) 2 (3%), 6 (8%) and 52 (68%), 16 (21%), 4 (5%), 0(0%), respectively. TIMP-2: tissue inhibitor of metalloproteinases-2; IGFBP-7: insulin-like growth factor binding protein-7

Twenty-four hours after CM infusion (*n* = 72 due to 2 discharges, 1 death, 2 missing samples), the median [TIMP-2]·[IGFBP-7] value was 0.10 [0.04; 0.37]. The median individual change in [TIMP-2]·[IGFBP-7] value from baseline to 24 h later was 0.00 [− 0.10; 0.09] (*p* = 0.40). Distribution of [TIMP-2]·[IGFBP-7] urinary concentrations with regard to various cut-offs at baseline, 6 and 24 h after CM infusion are presented in Fig. 2.

### Relationship between [TIMP-2]·[IGFBP-7] and the development of CA-AKI

Overall, KDIGO classification worsened within 72 h after CM infusion among 42 patients (55%), indicating possible CA-AKI. Eleven patients (14%) experienced severe AKI (KDIGO stage 3), two of whom underwent renal replacement therapy. In those 42 patients possibly developing CA-AKI, the median [TIMP-2]·[IGFBP-7] value at inclusion was 0.07 [0.03; 0.43]. No significant statistical association was observed between change in [TIMP-2]·[IGFBP-7] and CA-AKI occurrence. The median individual change was 0.01 [− 0.12; 0.24] and 0.20 [− 0.12; 0.20], respectively, 6 and 24 h after CM infusion (*p* = 0.29 and *p* = 0.88 respectively) in those patients.

Among patients not developing CA-AKI (stable KDIGO classification, *n* = 35), the median [TIMP-2]·[IGFBP-7] value at the time of inclusion was 0.06 [0.04; 0.11]. The median individual change was − 0.02 [− 0.08; 0.01] and − 0.01 [− 0.04; 0.06], respectively, 6 and 24 h after iodinated CM infusion.

Similar results were observed when defining the occurrence of CA-AKI as an increase in serum creatinine only (≥26 μmol/L), i.e., without using the urine output criterion of the KDIGO definition. Change in [TIMP-2]·[IGFBP-7] was − 0.02 [− 0.14; 0.03] and 0.03 [− 0.11; 0.35] at 6 and 24 h among the 14 patients (18%) developing CA-AKI according to the creatinine criteria only.

Only 13 patients (30%) possibly developing CA-AKI (worsening their KDIGO classification) had a significant (≥ 0.3) increase in [TIMP-2]·[IGFBP-7] value 6 h and/or 24 h after iodinated CM infusion. Conversely, among the 29 patients who had a significant (≥ 0.3) increase in [TIMP-2]·[IGFBP-7] 6 h and/or 24 h after CM infusion, 19 (66%) increased their KDIGO classification within 72 h (potential CA-AKI), a value close to the CA-AKI occurrence in the whole population of the study (55%). The 0.3 threshold of change in [TIMP-2]·[IGFBP-7] was not statistically associated with potential CA-AKI occurrence (*p* = 0.96).

### Influence of baseline renal function

As already ongoing AKI at the time of CM infusion may be an important confounder, we performed a subgroup analysis among the 42 patients without AKI at inclusion. In this subgroup, 10 patients (24%) had a clinically significant increase of [TIMP-2]·[IGFBP-7] (≥0.3) at 6 and/or 24 h after iodinated CM infusion; among those, 7 (70%) experienced a worsening of renal function within 72 h following iodinated CM infusion indicating possible CA-AKI. Conversely, among the 32 patients without AKI at inclusion and with no significant change in [TIMP-2]·[IGFBP-7] at 6 or 24 h, 14 (44%) patients experienced worsening renal function, reflecting a potential CA-AKI. However, this association between a significant increase in urinary [TIMP-2]·[IGFBP-7] and subsequent possible CA-AKI did not reach statistical significance (*p* = 0.29).

## Discussion

Among critically ill patients included in this study, CM infusion for an enhanced computed tomography did not induce significant changes in urinary [TIMP-2]·[IGFBP-7]. Among the subset of patients with an increase in [TIMP-2]·[IGFBP-7] considered clinically significant (≥0.3 at 6 or 24 h, *n* = 29 [38%]), we did not observe an association with possible CA-AKI, i.e., a subsequent worsening of renal function within 72 h of iodinated CM infusion defined according to the KDIGO classification [2, 18]. Those results are in line with recent epidemiologic studies showing a clinically negligible rate of AKI specifically attributable to iodinated CM [5–12]. Hence, practitioners who incorporate [TIMP-2]·[IGFBP-7] in their decision making process should not consider CM infusion neither as a significant cause of increased biomarker values nor as a major risk factor for development of AKI.

Our findings support the hypothesis of a clinically negligible toxicity of iodinated CM as used for enhanced-computed tomography. Indeed, there was no evident relationship between the increase in the sensitive urinary biomarker we used and the occurrence of a subsequent worsening of serum creatinine and/or urine output. In line with several recent epidemiologic studies, this suggests that, in AKI following an enhanced-computed tomography, the causal role of iodinated CM has been exaggerated for long [5–12]. Indeed, the multifactorial nature of AKI is particularly true in the setting of critical illness with multiple renal aggressions aside of CM. It is noteworthy that, in our study, when restricting analysis to the patients without ongoing AKI at the time of CM infusion (*n* = 42), only a minority (*n* = 10, 24%) experienced a significant increase in [TIMP-2]·[IGFBP-7] (≥0.3) at 6 or 24 h after CM infusion. However, among them, CA-AKI incidence was particularly high as 7 out of 10 developed CA-AKI. Conversely, CA-AKI incidence was lower, only 44%, among patients without ongoing AKI and not increasing [TIMP-2]·[IGFBP-7] after CM infusion. This result is in line with the high performance observed for cell-cycle arrest biomarkers in detecting true kidney aggression. Nonetheless, the difference between these incidences we observed in these subgroups did not reach statistical significance, possibly because of a lack of power.

Again, those results are potentially confirming the very limited kidney aggression induced by modern iodinated CM, aggression which is probably negligible compared to other causes of AKI among critically ill patients. However, specific pathophysiological pathways and measurement timing issues may be considered to explain the lack of association observed between CM infusion, change in urinary concentration of [TIMP-2]·[IGFBP-7] and CA-AKI. [TIMP-2] and [IGFBP-7] are both involved in G1 cell-cycle arrest during the very early phases of cell injury, a key phenomenon in septic and ischemic AKI [19, 20]. The Sapphire Study validated these biomarkers which outperformed other kidney aggression biomarkers among a large heterogeneous ICU population [13]. In the experimental setting, aside of direct osmolality-related tubular toxicity, pathophysiology of CA-AKI is believed to involve microcirculatory alterations and vasoconstriction resulting in medullar kidney hypoxia, oxidative stress and mitochondrial alteration [4]. As those pathways are common to most AKI etiologies, CM-induced increases in urinary [TIMP-2] and [IGFBP-7] would have been expected. However, one cannot rule out that human pathogenesis differs substantially from animal experimental models and that CM-induced kidney aggression predominantly occurs through G1 cell-cycle arrest independent pathways. Nevertheless, other biomarkers also showed inconsistent increases after CM infusion [21–23]. Very recent data explored the kinetics of cell cycle arrest biomarkers after various kidney aggression [24, 25]. Among 642 patients suffering various renal insults, Ostermann et al. observed a clear rise and fall in [TIMP-2]·[IGFBP-7] 24–48 h after major surgery and various toxic kidney aggressions (vancomycin, non-steroidal anti-inflammatory drugs, piperacillin-tazobactam). Interestingly no such change in [TIMP-2]·[IGFBP-7] was observed within 24–48 h among the 270 patients in which iodinated contrast media was the toxic kidney insult. Our results, showing a lack of change at 6 h and 24 h are complementary in the sense that an early and transient change in [TIMP-2]·[IGFBP-7] may have been missed by the study design of Ostermann et al. [24].

### Study limitations

Patients in this study received various iodinated CM, mainly iohexol, iobitridol and iopromide (Table 1), results generalizability to other low osmolarity CM may warrant specific studies.

Timing of urinary measurements in the present study may have been inadequate to show a CM-induced increase in [TIMP-2]·[IGFBP-7] [24]. However, a [TIMP-2]·[IGFBP-7] increase later than 24 h after CM infusion, is unlikely as values rather dropped from 6 to 24 h after CM (Fig. 1). Furthermore, a late diagnostic biomarker would be of little clinical value. Conversely, changes in urinary metabolites occur very early after CM infusion [26]. Thus, an early and transient [TIMP-2]·[IGFBP-7] increase (< 6 h) may have been missed by the study design. However, such a short-lasting aggression would rather signify relative innocuity of CM compared to other mechanisms of AKI associated with sustained [TIMP-2]·[IGFBP-7] increases.

A combined analysis of [TIMP-2]·[IGFBP-7] was performed using the point-of-care Nephrocheck® measurement device which may be seen as a limitation. Indeed, different performances of each component, such as observed in other settings, with TIMP-2 outperforming IGFBP-7 for sepsis-induced AKI diagnosis and IGFBP-7 being superior in the surgical setting, could not be evaluated [13, 27]. These differences may underline subtle but important mechanistic differences between various etiologies of AKI, and the different pathways of kidney cell injury. Unfortunately, separate values for TIMP-2 and IGFBP-7 could not be measured in our study.

Inclusion of a control group of patients not receiving CM would have enabled to clearly delineate CM attributable increases in [TIMP-2]·[IGFBP-7]. However, at best, changes in [TIMP-2]·[IGFBP-7] would have been similarly insignificant in a control group given the results of the present work.

Our study was not designed to assess the performance of [TIMP-2]·[IGFBP-7] for the early detection of CA-AKI. Using a sensitive biomarker, this study rather aimed at addressing the issue of the clinical relevance of the toxicity of iodinated CM in the critically ill with a stand point complementary of epidemiologic studies [5–12]. However, despite robust validation studies [13, 14], one cannot definitely rule out a lack of sensitivity of [TIMP-2]·[IGFBP-7] to explain our findings. The 0.3 cutoff value chosen to define a significant increase in [TIMP-2]·[IGFBP-7] after CM infusion has been only proposed previously as an absolute value to assess the risk of AKI, in studies not specifically addressing CA-AKI [16]. The median change in [TIMP-2]·[IGFBP-7] was similar among patients worsening (55%) and patients not worsening their renal function after exposure to CM (45% of patients), therefore, as suggested by Fig. 1, the choice of a different cutoff would not impact our findings. Nevertheless, given the numerous confounding factors which may contribute to AKI, results should be interpreted cautiously.

Last, since CM infusion did not induce significant changes in urinary [TIMP-2] ·[IGFBP-7], lack of power may be a contributing factor. The study was powered to detect a 0.3 increase in [TIMP-2]·[IGFBP-7], one cannot exclude that smaller but statistically significant increases may be detected by a larger study, which may also enable to perform several complementary subgroup analyses.

## Conclusion

Changes in urinary concentration of [TIMP-2]·[IGFBP-7] induced by contrast-enhanced computed tomography were insignificant. Those results are compatible with the hypothesis that kidney aggression induced by modern iodinated CM is minimal.

## Abbreviations

AKI: Acute Kidney Injury
CA-AKI: Contrast-associated acute kidney injury
CM: Iodinated contrast media
IGFBP-7: Insulin like growth factor binding protein 7
KDIGO: Kidney Disease Improving Global Outcome
TIMP-2: Tissue inhibitor of metalloproteinase 2
